# Patient Satisfaction with Health Care Services; An Application of Physician’s Behavior as a Moderator

**DOI:** 10.3390/ijerph16183318

**Published:** 2019-09-09

**Authors:** Faiza Manzoor, Longbao Wei, Abid Hussain, Muhammad Asif, Syed Irshad Ali Shah

**Affiliations:** 1 Department of Agricultural Economics and Management, School of Management, Zhejiang University, Hangzhou 310029, China or (F.M.) or (S.I.A.S.); 2 School of Public Affairs, Zijingang Campus, Zhejiang University, Hangzhou 310029, China or (A.H.) or (M.A.)

**Keywords:** patient satisfaction, healthcare services, laboratory and diagnostic care, preventive healthcare, prenatal care, physician’s behavior, Pakistan

## Abstract

Patient satisfaction is a measure of the extent to which a patient is content with the health care they received from their health care provider. Patient satisfaction is one of the most important factors to determine the success of a health care facility. The purpose of this study was to determine patient satisfaction with healthcare services and encompass the physician’s behavior as moderation between patient satisfaction and healthcare services. The study seeks to measure the health care services, like a laboratory and diagnostic care, preventive healthcare and prenatal care, to patient satisfaction in the public health sectors of Pakistan. A descriptive survey research design was used for this study. The target population was patients from the out-patient department (OPD) of three public hospitals from Pakistan. By using the convenient sampling technique, 290 sample participants were selected from the target population. The reliability scales were tallied by using Cronbach’s Alpha. The findings of the study are gleaned by using regression to explore patient satisfaction with the health care services, and whether or not the physician’s behavior moderates the link of patient satisfaction and healthcare services. SPSS Hayes process was used for the moderation effect of the physician’s behavior. The main results of the regression analysis validate that health care services, such as laboratory and diagnostic care, preventive healthcare, and prenatal care, have a significant and positive effect on patient satisfaction. Specifically, the study suggests that the physician’s behavior significantly moderates the effect of health care services on the satisfaction of patients. The overall opinions about the satisfaction level of patients for the availability of health services in the hospitals were good. The degree of satisfaction was satisfactory with respect to laboratory and diagnostic care, preventive healthcare, and prenatal care services. Based on the outcomes, the study confirms that the proposed hypotheses are statistically significant. Furthermore, the directions for future research of the study are offered.

## 1. Introduction

Global competition on an emerging sector drives the curiosity of patients and makes them more anxious towards the delivery of healthcare services. The growing concern about health and elevated economic levels of modern civilization have intensely improved the healthcare demands and shifted trends of the population towards attaining a healthier lifestyle [1]. An expansion in the global competition for the provision of services has created a rigorous situation that influences the inhabited business, particularly medical services. As a result, the health care relationship has begun to stress on the superior healthcare service delivery due to the growing competition among hospitals. This delivery has persuaded the patients to make the best choice in selecting any hospital [2].

Improved patient care has become a priority for all health care service providers with the optimum objective of achieving a high degree of patient satisfaction [3]. At the same time, good healthcare service delivery, as compared to their counterparts, provides businesses or public trusts with the opportunity to distinguish their facilities in a competitive industry [4]. Currently, on account of the expanded expectations for ordinary services and higher customer’s needs, it is obligatory for hospitals to give superior health care services to the patients and to fulfill their requirements [5]. In previous decades, healthcare services and their services are one of the rare topics in service studies in developing countries like Pakistan. While it has received extensive academic study, the need for improvement in healthcare services has grown which leads to challenges for the service provider (i.e., technical or non-technical staff) and has become a complex task for scholars, government policymakers, therapeutic specialists and hospital administrators to fulfill the requirements of clients which help toward developing satisfaction [6].

Satisfaction is one of the key factors pertaining to government policy or a successful business which can only be sustained through the delivery of exquisite service quality resulting in improved satisfaction. These ameliorated provisions require effective service delivery, cost allocation, and management strategies [7]. In the context of suppliers, there are two forms of service providers that are working particularly well in developing countries in both the private and public sector hospitals. Selecting the right health center and skilled physician is imperative to fulfil the aim of patient satisfaction as it suggestively influences the treatment of the patient [8].

The patient’s opinion is becoming more important in the improvement process of a health care delivery system. Patient satisfaction is the state of pleasure or happiness that the patients experience while using a health service. Thus, patient care is the basic function of every health service provider [9]. It is one of the standards to measure the efficiency and effectiveness, where the efficiency of a hospital is associated with the provision of service delivery and quality care. Patient satisfaction is the actual evidence of the effectiveness of the healthcare services providing administration [10].

Patient satisfaction is a renowned standard to evaluate the effectiveness of health services being provided in hospitals. Patient satisfaction is an important measuring stick by which the delivery of health care service is the measure [11]. Currently, the patients’ opinions are considered as a key factor in the decision of treatment and delivering health care services [12]. Hence, the evaluation of health service delivery from the patients’ perspective has received greater attention and has become a core attribute of any health system as it serves as a valuable indicator to measure the success of a service provision, especially in public sector hospitals [13].

The public sector hospitals work under the government policies because the government only funds public sector hospitals, while the private sector organizations are established as business organizations that could provide more effective care and services to their clients. The private hospital patients require paying more money in order to get the desired service quality. At present, for the patient’s demands, exact and complete details are required before using any kind of services by a particular health care organization. The patients have become more inquisitive and expect supplementary services to gain the quality of services beyond their expectations, because they are paying more money for treatments, and simply any occurrence of dissatisfaction tends to force them to move towards other competitors [14]. However, the quality of service delivery is considered as an essential factor in promising general patient satisfaction towards hospitals. It has been suggested that physicians and hospital staff (medical or non-medical) all ought to focus on the direction to improve as well as enhance the quality of service delivery [15].

Manimay [16] proposed that giving powerful training to service providers (including all staff of the hospital, medical or non-medical) on interpersonal skills and realistic communication stimulates patient satisfaction. In hospitals, the enhancement of service quality is compulsory for achieving a trustworthy profile for patients. Zeithaml [17] indicated that at the time when service delivery was assenting for the assessment of services, it affected the patients’ attitude and expectations which, in turn, strengthened their relationship with the providers. As a result, the procedure of service delivery has had a great impact on the management of all the involved services and organizations which ought to be especially concerned with providing detailed medical attention. In addition, it is important to focus on supporting the best service quality to the public for satisfaction.

As per the literature, this aspect of health care has been totally ignored in Pakistan. Prenatal care and preventive care are essential factors for the many women who face issues during pregnancy, as well as some of them dying due to the lack of facilities. It has been indicated in the above discussion that the essential responsibilities of service providers are to build and maintain patient satisfaction as well as providing better service delivery with significant medical care by the desirable services as distinguished by the citizens. This might be practicable if hospital staff (technical or non-technical) are able to understand and know the patients’ perceptions.

A physician’s behavior is also a main component of patient satisfaction. In public sector hospitals, there are numerous patients, heavy workloads for staff, and a work environment where physicians/doctors can behave rudely. To some extent, the patient’s repeated silly questions only need common sense to answer rather than medical knowledge. Whenever confronted with such types of situations, doctors tend to give a rude reply [18]. On the other hand, a physician’s medical knowledge is very important for the care for patients, and unique clinical best practices of specialties would be a major determinant for the patient’s medical use [19]. Many studies showed that patients expect to have a comfortable and warm interaction with a physician who appears to be technically competent and gives adequate information about the illness [20,21]. In developing and developed countries, many studies have been conducted to measure patient satisfaction with healthcare services [22,23]. This study assesses patient satisfaction with health care services (laboratory and diagnostic care, preventive healthcare and prenatal care) in Pakistani public hospitals. The main reason for choosing these services is that no previous study has been conducted regarding these services in the country. There is a considerable gap in existing literature regarding this evaluation of patient satisfaction, which has been often ignored in empirical research. The current study is specifically useful for the assessment of the Pakistani health care system because the system is commonly allied with a lack of patient satisfaction and quality of life. Based on the literature, the authors found no such type of study in Pakistan concerning patient satisfaction, especially with respect to the following healthcare services, such as laboratory work with diagnostic care, preventive healthcare, prenatal care and physician behavior as a moderator.

The rest of this article is structured as follows: The second section shows the literature and hypotheses development. Then, the methodological framework is described in the third section. In the fourth section, the results of the study and the interpretation of the results are discussed. At last, the outcomes of the study are examined. In the discussion, the implications of the study and some suggestions for future research directions are also presented.

## 2. Review of Literature and Hypotheses

### 2.1. Healthcare Service and Patient Satisfaction

Best healthcare service delivery empowers hospital management to differentiate the hospital and upgrade proficiency and increase a practical competitive favorable position. Gronroos [24] characterized perceived quality as an assessment procedure, where the customer compares their expectations with their service observations. The quality of healthcare service is the disparity among customer perceptions and their assumptions regarding services. In the healthcare setting, the patients are the capital of the hospital. To satisfy and sustain customers, medical service delivery has turned out to be reasonably more imperative [25]. The research addressing the patient’s judgment conducted in developed states revealed that patients can evaluate the procedure of services, results, and structure [26]. The studies have elaborated the association between patient satisfaction and healthcare service delivery [27,28]. Cronin Jr and Taylor [29] concluded that there is a strong relationship between healthcare service delivery and satisfaction. Additionally, Badri et al. [30] recognized that the patients are identifying the important points in the hospital, being the appraisal and execution of health services. In addition, addressing the needs of patients and medical service standards are essential towards achieving high significance. In the settings of health services, a client’s satisfaction is generally used to assess the quality of service delivery.

Shabbir et al. [8] and Asif, et al. [31] indicated that there is an association between patient satisfaction and healthcare service quality. The findings of their studies showed a significant connection between patient satisfaction and healthcare services. Patient satisfaction is also determined by exploring the particularity between the expected and perceived health services. Effective public learning can establish trust which can give significant appraisal to the hospital administration [32]. Chahal and Mehta [33] and Naidu [34] described how healthcare service delivery influenced patient satisfaction. Patient satisfaction assists as a mode between behavioral intentions and the quality of healthcare service delivery.

### 2.2. Laboratory and Diagnostic Care

Currently, laboratory services are considered as the backbone in the healthcare sector. The world is rapidly progressing in the technology industry due to the number of diagnostic machines found in the laboratory that have saved millions of people’s lives, such as advanced ultrasound, magnetic resonance imaging (MRI), pathology tests and much more advancement in testing [35]. Laboratory services are essential for assisting in the diagnostic diseases of patients because in several cases, the physicians know the severity of the patients’ illness by laboratory services. Laboratory services with diagnostic care and patient satisfaction have a strong relationship. Particularly, in prenatal services for the examination of pregnant women, as well as for the examination of baby conditions [36].

Therefore, several scholars consider it an important element in healthcare services, as well as in patient satisfaction [37,38]. Abera et al. [39] found that laboratory services impact significantly on patient satisfaction, while, Kamra et al. [40] indicated that through laboratory services, hospital management can improve patient satisfaction. Wankar [41] described it as a vital factor for the prenatal services in the hospitals. This study addresses the actual conditions of this factor on female satisfaction or other patients in Pakistan. The laboratory role, as a diagnostic tool for illnesses, is very significant in healthcare for the public as any other practice of medical services. Additionally, no one can deny the importance of laboratory services in healthcare. This is clear by the World Health Organization (WHO) when they incorporated laboratory services in healthcare [42]. Therefore, the WHO recommended to country members to promote and develop healthcare services through the laboratory of health under the primary healthcare approach [43].

### 2.3. Preventive Healthcare

The implementation of good practice preventive care has the potential to significantly enhance healthcare results by lessening the prevalence of amenable risk aspects. The services of healthcare are well placed to address the issues of chronic disease prevention and administration [44], with every medical care visit being a potential chance to provide preventive care [45].

Prevention is identified by both the patients and physicians as one of the basic tasks of a physician [46]. The efficiency of brief involvement (described as brief, patient-centered relations and motivational) of physicians in hopeful changes in smoking, alcohol, weight, and physical activities behavior has been demonstrated [47].

### 2.4. Prenatal Care

Prenatal care is the most essential factor all over the globe. Prenatal care connects to any condition or event that exists or occurs at the beginning of the pregnancy from the time it starts to infant delivery. Therefore, prenatal care is linked with the doctors, nurses or midwives’ behavior, as well as the behaviour of technical staff, throughout the pregnancy examination until the infant’s healthy birth [48].

The services of prenatal care, which entail directly supervising the well-being of the unborn baby and mother, have been seen as potentially one of the most effective health interventions for preventing maternal morbidity and mortality [49,50]. Childbirth and pregnancy issues are leading death problems, particularly for women with disabilities at reproductive age, mostly in developed nations [51]. The health professionals in the hospitals directly perform prenatal and postnatal services with all pregnant women, as well as recommend or make the patients aware of the schedule for the next visits for the examinations [52].

Several scholars use this variable for the study of patient satisfaction because prenatal is a primary element in the healthcare sector for evaluating service delivery. Pregnant ladies prefer hospitals which provide better facilities, such as medical tests, physical examination,, and knowledge about conditions with friendly behaviour from the doctors [53,54]. Therefore, a number of researchers identified that prenatal care impacts on patient satisfaction [55,56]. The effective service of prenatal care is not the only purpose of the hospital, but also improving the level of satisfaction with health services. The information about the customer’s views is still very particular in developed states [48].

### 2.5. Physician’s Behaviour

“A physician shall uphold the standards of professionalism, be honest in all professional interactions, and strive to report physicians deficient in character or competence, or engaging in fraud or deception, to appropriate entities” [57]. The physician’s behaviour is a key factor for patient satisfaction where the physician-patient interactions depend, at least partly, on how physicians interpret and respond to patients. Robin DiMatteo, et al. [58] revealed that patients expect good relationships and polite behavior from their physicians. It also affects the patients’ decision to remain committed to their physicians. Several authors have suggested that when these expectations are disappointed, patients are less satisfied and less likely to comply with their medical regimen, return for appointments, or otherwise cooperate in their own treatment [59,60]. Korsch and her associates have performed several studies of the relationship between what occurs in the physician-patient interaction and subsequent patient satisfaction and compliance [61,62]. As her studies were performed in a pediatric outpatient clinic, compliance and satisfaction were attributed to the patient’s parent rather than the patient. In general, the greater the friendliness and solidarity expressed by the physician, the more satisfied and more complaint was the patient. These studies as well as others [63] have all found that information-giving by the physician and his/her polite manner is positively correlated with patient satisfaction. The past literature showed that studies regarding the physician’s behaviour are limited. Therefore, to fill this gap, there is strong need to explore the abovementioned relationships between the study variables.

The major objective of this research is to examine patient satisfaction with health care services with the moderator role of the physician’s behavior. After a careful review of the literature, the authors have formulated the following hypotheses:

**Hypothesis** **1** **(H1).** Laboratory and diagnostic care are positively associated with patient satisfaction.

**Hypothesis** **2** **(H2).** Preventive healthcare has a positive association with patient satisfaction.

**Hypothesis** **3** **(H3).** Prenatal care has a positive correlation with patient satisfaction.

**Hypothesis** **4** **(H4).** There is a positive relation between the physician’s behavior and patient satisfaction.

**Hypothesis** **5a** **(H5a).** The physician’s behavior has a positive moderating relationship between laboratory and diagnostic care and patient satisfaction.

**Hypothesis** **5b** **(H5b).** The relationship of preventive healthcare and patient satisfaction is moderated by the physician’s behavior.

**Hypothesis** **5c** **(H5c).** The relationship of prenatal care and patient satisfaction is moderated by the physician’s behavior.

## 3. Methods

### 3.1. Study Setting

The present study was conducted at the public health sector of Khyber Pakhtunkhwa (abbreviated by KPK), Pakistan. KPK covers 101,741 km^2^. KPK is the third largest province in the country by the size of both the population and the economy [64].

### 3.2. Hypothesized Model

**Figure 1:**
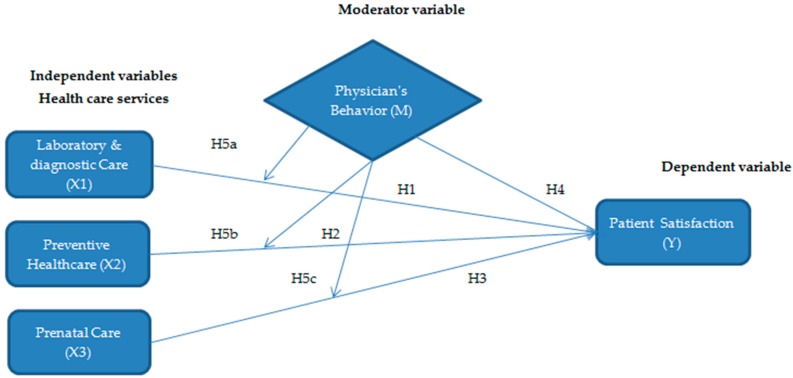
Conceptual (Hypothesized) model.

The main purpose of this study is to determine patient satisfaction with healthcare services with the inclusion of the physician’s behavior as a moderator variable in public hospitals of KPK, Pakistan. Reflecting on the literature and the postulated hypotheses, the conceptual model of the study has been developed, as exhibited in Figure 1. The model depicted in this study states that better health care services, such as laboratory and diagnostic care, preventive healthcare, and prenatal care improve patient satisfaction (Hypotheses 1–3 respectively). The good and soft physician’s behavior has a positive link to patient satisfaction (Hypothesis 4). Furthermore, the physician’s behavior also moderates the relation of healthcare services and patient satisfaction (Hypotheses 5a–c). The hypothesized study model is presented in Figure 1.

### 3.3. Participants and Data Collection Procedure

The participants who participated in the current study were OPD patients from the three public hospitals in Pakistan. These hospitals were King Abdullah Hospital Mansehra, Ayyub Teaching Hospital Abbottabad, and Khyber Teaching Hospital Peshawar. In this study, the participation of the patients was voluntary. The present study used self- administrated questionnaires for primary data collection from the participants. One questionnaire was constructed which consisted of all the study variables and the survey was conducted from early June to late July 2018. These questionnaires were originally developed in English, and after that, translated into the Urdu (national language) for the better understanding of the local people. The authors personally visited the hospitals and all of the respondents (patients) were told about the aim of the study and they were encouraged to contribute. Furthermore, the authors guaranteed the privacy of the responses of the participants. However, the face-to-face interviews were also conducted in the case of willing patients with no education or low education as well as due to the shortage of time.

By using the convenient sampling technique, originally 350 questionnaires were distributed to the people, and only 320 filled questionnaires were returned, out of which 30 cases had to be excluded due to missing data for different variables.

### 3.4. Frequency Analysis

A frequency analysis was used for the demographic data analysis and general information about the survey participants. The results of the frequency analysis or demographic assessments are presented in Table 1. This includes age, gender, education, occupation, background, and marital status of the participants. The majority part of the participants were women (55.2%). Many of them were in the 26–29 year-age group. The larger part of respondents had no education (24.1%). Further, 195 of the respondents belonged to the rural area and most were married (62.1%). Regarding the respondent’s occupation, a larger proportion were servants of the government.

### 3.5. Measurement Instruments and Cronbach’s Alpha Reliability

During this study, a number of measurement instruments have been used. These instruments are described below. The internal consistency was evaluated by calculating Cronbach’s alpha reliability coefficients for each scale. An alpha coefficient of greater than 0.7 was considered acceptable [65,66].

This study adopted a 10-items laboratory and diagnostic care (LDC) scale from Almatrafi et al. [67]. An example item for LDC is “Accessibility of phlebotomy room?” The alpha coefficient was 0.79 for this scale. The preventive healthcare (PHC) scale with 6 items was taken from Parchman et al. [68]. A sample item for PHC is “Are you satisfied with your follow-up care?” The alpha value for PHC was 0.94. The prenatal care (PC) scale consists of 8 items adopted from Sword et al. [69] with a sample item “I received adequate information about my diet during pregnancy?” The alpha for this scale was 0.82. This study employed a 9-item physician’s behavior (PB) scale developed by Wolf et al. [21]. Its sample item is “The physician gave me a thorough check-up”. Cronbach’s alpha for this scale was 0.81. A 9-item patient satisfaction (PS) scale was taken from Soomro et al. [70] with a sample item of “Are you satisfied with the services of paramedical staff”. Cronbach’s α for PS was 0.71. All items were measured using a 5-point Likert scale. The α reliabilities were above the cutoff value of 0.70 [71,72].

## 4. Results

### 4.1. Descriptive Statistics

The mean scores, standard deviations, correlation and alpha reliabilities of all variables are depicted in Table 2. All health care services are positively correlated. Patient satisfaction, like laboratory and diagnostic care, has a positive correlation with (r = 0.260); preventive healthcare has a positive correlation with (r = 0.347); prenatal care has positive correlation with (r = 0.438) and physician’s behavior has a positive correlation with (r = 0.251) to patient satisfaction.

### 4.2. Multiple Regression Analysis and Results Interpretation

To test the main hypotheses for the present study, the multiple linear regression was used (IBM SPSS v.25) [73]. Multiple regression is an extension of simple linear regression [74]. It is used to predict the value of an outcome variable (dependent variable) based on the value of two or more predictor variables (independent variables) [75,76]. For analysis, the mean value of the variables was used. The results in Table 3 reveal that health care services have a positive and affirmative effect on the predicted variable. The results reported in Table 3 that laboratory and diagnostic care have a positive and significant relationship to patient satisfaction with (β = 0.117, *p* = 0.007). Therefore, Hypothesis 1 is accepted and the null hypothesis is rejected. Similarly, preventive healthcare services and patient satisfaction have a positive association with (β =0.202, *p* = 0.000). However, the null hypothesis is rejected and alternate Hypothesis 2 is statistically accepted. Further, prenatal care and patient satisfaction have a positive and significant correlation with (β = 0.260, *p* = 0.000). Hence, the results are supporting Hypothesis 3 and here, the null hypothesis is rejected. Furthermore, the physician’s behavior with the value of (β = 0.107, *p* = 0.02) has a significant and positive effect on patient satisfaction. Therefore, these findings reject the null hypothesis and support Hypothesis 4. Eventually, the outcomes showed that the overall regression model is significant.

Furthermore, using a Bonferroni correction, patient satisfaction is associated with health care services with (F = 78, *p* = 0.03).

### 4.3. Moderation Analysis

For the present study, physician’s behavior is used as a moderator variable. A moderator variable, commonly denoted as W, is a third variable that affects the strength of the relationship between a predicted and predictor variable in a correlation [77]. A moderation analysis is a kind of regression analysis which explains the impact of the independent variable on dependent variable through or under the influence of a moderator, which is a third variable [78,79]. This study used Hayes process (v. 3) [80] by the computer software IBM-SPSS (v. 25) to validate the moderation hypotheses for a recent study. For the direct relationship of health care services (i.e., laboratory and diagnostic care, preventive healthcare, prenatal care) and patient satisfaction, multiple regression techniques were used, and to check the moderation effect of the physician’s behavior on these predictor variables and the predicted variable, Hayes process moderation analysis was used. Hayes process is considered an effective and more powerful process than its alternatives [81], and 5000 bootstrapping-based resample has been chosen. Bootstrapping does not have any assumption of normal distribution.

Table 4 demonstrates the outcomes of Hayes process [80] moderation (interation1, interaction2, and interaction3) are discussed about the moderation effect of the physician’s behavior between healthcare services and patient satisfaction. In Hypothesis 5a, the recent study anticipated that interaction1 (LDC *PB) has a significant effect on patient satisfaction with the 0.009 *p*-value. These findings support Hypothesis 5a. Interaction2 (PHC *PB) also has a significant effect on patient satisfaction. In Table 4, the results of interaction2 showed *p*-value is 0.03, so Hypothesis 5b is accepted. Furthermore, in interaction3 (PC *PB), the outcomes reveal that interaction3 has a significant effect on a predicted variable with the *p*-value of 0.000 which is less than 0.05. Hence, these results fully support Hypothesis 5c. Therefore, the findings of the moderation analysis yielded that there is a significant moderating effect of the physician’s behavior on healthcare services and patient satisfaction.

### 4.4. Interpretation of Plotting

**Figure 2:**
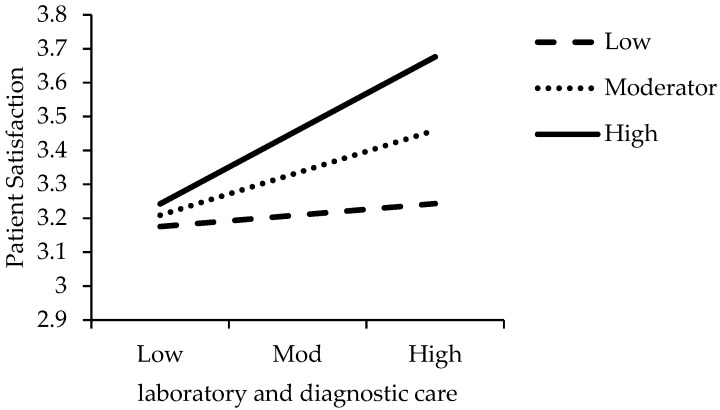
Interactive effect of the laboratory and diagnostic care (LDC) and physician’s behavior (PB) on patient satisfaction.

**Figure 3:**
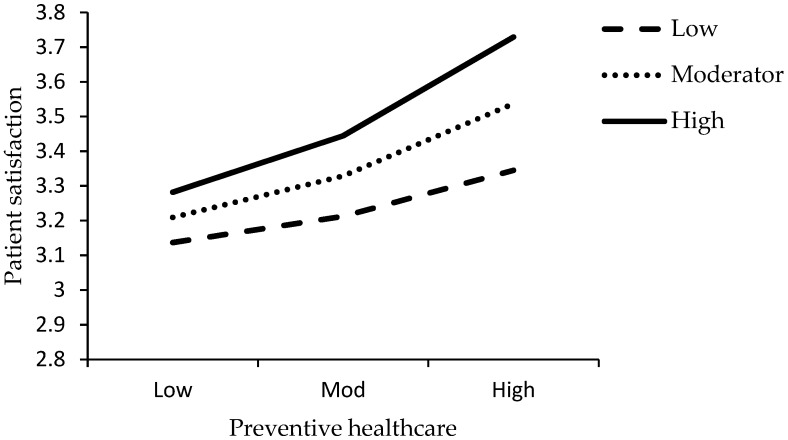
Interactive effect of the preventive healthcare (PHC) and PB on patient satisfaction.

**Figure 4:**
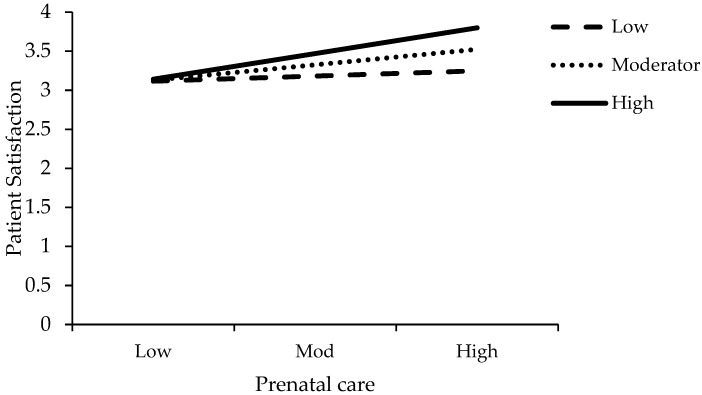
Interactive effect of the prenatal care (PC) and PB on patient satisfaction.

This study plotted the interaction graph for low, moderator and high values. Figure 2 shows the interactive effect of the LDC and PB on patient satisfaction. Figure 3 shows the interactive effect of the PHC and PB on patient satisfaction. Figure 4 shows the interactive effect of the PC and PB on patient satisfaction.

## 5. Discussion

The main objective of the present study is to determine patient satisfaction with healthcare services with an application of physician’s behavior as a moderator role. This study has been conducted in public hospitals in Pakistan. Three health care services were chosen, such as laboratory and diagnostic care, preventive healthcare and prenatal care, to determine the level of patient satisfaction. Furthermore, the study examined the physician’s behavior as a moderating role in the relationship between healthcare services and patient satisfaction.

This study provides knowledge and contribution to health care literature. While trawling through the literature, it was evident that many of the studies have been conducted in emerging, developing and developed countries [82,83,84,85]. Prior studies demonstrated patient satisfaction in mental health care [86], as well as cancer patient satisfaction [87]. This study has the main focus of laboratory and diagnostic care, preventive healthcare and prenatal care to patient satisfaction, and this study has the novelty of the moderating role of the physician’s behavior in developing countries, like Pakistan. The findings of the present study showed that healthcare services and patient satisfaction are positively and significantly associated with each other. For instance, the predictor variable, healthcare service laboratory and diagnostic care, are positively and significantly associated with the predicted variable of patient satisfaction. The findings from this research support a previous study highlighting the assessment of customer satisfaction with the clinical laboratory services provided in King Abdullah Medical City, Makkah [67]. Furthermore, the outcomes of preventive healthcare and prenatal care have a positive and significant association to patient satisfaction. The findings of preventive healthcare and patient satisfaction are supported by the previous study by Jayanti and Burns [88] and Parchman [68]. The results of prenatal care and patient satisfaction are in the line with the earlier research of Oladapo et al. [89] which demonstrated that the majority of the women expressed satisfaction with the level of expertise and basic technical competence of their care providers. In addition, the present study revealed that the moderator variable of the physician’s behavior is also positively and significantly correlated with the predicted variable of patient satisfaction. These results are consistent with the findings of the previous study of Taenzer et al. [90] who posited that the physician’s behaviour is closely related to patient satisfaction. The main outcomes are fully supported by the hypotheses that there is a positive association among healthcare services and patient satisfaction.

Many scholars have investigated the affirmative and optimistic association among healthcare services and patient satisfaction [27,28]. The study examined the moderating role of the physician’s behavior between healthcare services and patient satisfaction, which is nearly non-existent and very limited. However, the current study examined this gap and confirmed that the physician’s behavior has an optimistic and positive moderating influence in the correlation among healthcare services (laboratory and diagnostic care, preventive healthcare, and prenatal care) and patient satisfaction. Furthermore, the findings of the moderation analysis revealed that the assumed hypotheses are fully acceptable.

The theoretical contributions of the current study are multifold. First, it has a contribution to the emerging field of healthcare services in developing countries by investigating how this notion can function in the health sector. This study also addresses the neglected link in research between healthcare services and satisfaction. Second, a large part of this research on the association between healthcare services and patient satisfaction demonstrates that patients feel better due to the best services. For physicians and hospital staff, the findings show that providing best and quick services is key for the satisfaction of patients. Consequently, it is beneficial for patients as well as staff. The practical contribution of the current study is patient satisfaction with health care services, that how to provide best services to improve patient satisfaction. Therefore, providing the best health services plays a main role in boosting patient satisfaction. The findings of this study are encouraging healthcare organizations to boost their service delivery. Hopefully, this study will play a significant role in the literature of the healthcare sector. Furthermore, the government should pay more attention to the maintenance level of the healthcare sector of the country.

As with any study, this research has a few limitations, which suggest several questions for future study. First, the primary data was collected from the outpatient department for this study. A future study can use data from the inpatient care department (admitted patients). Second, the current study is limited to three hospitals in the context of one province of Pakistan. Future studies could be conducted in the other hospitals of remaining provinces of Pakistan. Furthermore, this study is limited to one developing country. Future studies are highly recommended to other developing and emerging countries. The existing study used four healthcare services. Future studies are thus encouraged to investigate other healthcare services, such as transplantation services, dental services and heart/cardiovascular Services, etc. Lastly, the present research examines the stated questions from the patient’s perspective. Future studies are encouraged to examine whether physicians are satisfied with the facilities of hospitals or not.

## 6. Conclusions

The present study aimed to investigate patient satisfaction with healthcare services in the context of Pakistan. In developed countries, many studies have been conducted about health care services and patient satisfaction, but there has been less attention to developing countries like Pakistan. This study identifies the relationship between healthcare services (laboratory and diagnostic care, preventive healthcare, and prenatal care) and patient satisfaction with the moderating role of the physician’s behavior. The current study was conducted in the three public sector hospitals namely; King Abdullah Hospital Mansehra, Ayyub Teaching Hospital Abbottabad, and Khyber Teaching Hospital Peshawar, Pakistan. In this study, health care services, such as laboratory and diagnostic care, preventive healthcare, and prenatal care, are explanatory variables and patient satisfaction is the outcome variable, and the physician’s behavior is used as a moderating variable. It was finalized from the present study that there is a positive effect of health care services on patient satisfaction.

The findings of the study showed that patients were satisfied with the efficiency of services. Patient satisfaction and healthcare services (laboratory and diagnostic care, preventive healthcare, and prenatal care) have a positive and significant association. Further, the physician’s behavior has moderated among healthcare services and patient satisfaction. Therefore, the alternate hypotheses are accepted.

It is concluded that better and best healthcare services play a crucial role in patient satisfaction. Healthcare centers and public hospitals need to improve their facilities and make better services in developing countries. Poor people visit public hospitals, and their satisfaction is very important. There is a need for doctors/physicians to be polite, empathetic and concerned with their patients. They should behave politely with the patients and their attendants. Physicians should give them a thorough check up and examination, treat them with courtesy and respect. Therefore, in this sector, the working conditions and surroundings are not healthy, and the workload and numerous patients are solely responsible for the physician/doctors’ rudeness. However, in public sector hospitals, governments should focus on hiring additional staff to overcome the workload.

## Figures and Tables

**Table 1:** Demographic assessments.

**Gender**	**Frequency**	**Percent**	**Age**	**Frequency**	**Percent**	**Education**	**Frequency**	**Percent**
Male	130	44.8	20–25	35	12.1	No education	70	24.1
Female	160	55.2	26–29	80	27.6	Elementary school	60	20.7
Total	290	100	30–39	60	20.7	High school	60	20.7
			40–49	50	17.2	Bachelor/College	45	15.5
			50 above	65	22.4	Master degree	55	19
			Total	290	100	Total	290	100
**Occupation**	**Frequency**	**Percent**	**Marital Status**	**Frequency**	**Percent**	**Backg-round**	**Frequency**	**Percent**
Govt. servant	130	44.8	Married	180	62.1	Rural	195	67.2
House wives	70	24.1	Single	45	15.5	Urban	95	32.8
Retired	30	10.3	Divorced	5	1.7	Total	290	100
Farmers	40	13.8	Widow	60	20.7			
Students	20	6.9	Total	290	100			
Total	290	100						

**Table 2:** Descriptive statistics, correlations, and reliability.

Variable	Mean	SD	1	2	3	4	5
1. Laboratory and diagnostic care	3.680	0.671	**0.79**				
2. Preventive healthcare	2.491	0.971	0.047	**0.94**			
3. Prenatal care	3.324	0.851	0.202 **	−0.007	**0.82**		
4. Physician’s behavior	3.991	0.601	0.268 **	0.018	0.252 **	**0.81**	
5. Patient Satisfaction	3.352	0.576	0.260 **	0.347 **	0.438 **	0.251 **	**0.71**

** Correlations are significant at the 0.01 level (2-tailed). Bold values reveal α coefficient. SD: standard deviation.

**Table 3:** Multiple regression.

Variables	β	T	Significance	95% Confidence Interval	
				Lower Bound	Upper Bound
Constant	1.130 ***	4.969	0.000	0.682	1.578
Laboratory and diagnostic care	0.117 **	2.728	0.007	0.033	0.202
Preventive healthcare	0.202 ***	7.131	0.000	0.146	0.258
Prenatal care	0.260 ***	7.716	0.000	0.194	0.327
Physician’s behavior	0.107 *	2.201	0.029	0.011	0.202

Dependent variable: Patient Satisfaction (PS), * *p* < 0.05, ** *p* < 0.01, *** *p* < 0.001.

**Table 4:** Moderation analysis.

Interactional Effect	β	SE	T	Sig.	95% Bootstrapping Confidence Interval	
					LLCI	ULCI
Interaction1 (LDC *PB)	0.226	0.086	2.617	0.009	0.056	0.397
Interaction2 (PHC *PB)	0.117	0.056	2.063	0.031	0.0054	0.229
Interaction3 (PC *PB)	0.256	0.050	4.836	0.000	0.1518	0.360

SE = standard error; LLCI = lower limit confidence interval; ULCI = upper limit confidence interval.
